# Cost–benefit of limited isolation and testing in COVID-19 mitigation

**DOI:** 10.1038/s41598-020-75640-2

**Published:** 2020-10-29

**Authors:** Andreas Eilersen, Kim Sneppen

**Affiliations:** Niels Bohr Institute, University of Copenhagen, 2100 Copenhagen, Denmark

**Keywords:** Epidemiology, Numerical simulations

## Abstract

The international community has been put in an unprecedented situation by the COVID-19 pandemic. Creating models to describe and quantify alternative mitigation strategies becomes increasingly urgent. In this study, we propose an agent-based model of disease transmission in a society divided into closely connected families, workplaces, and social groups. This allows us to discuss mitigation strategies, including targeted quarantine measures. We find that workplace and more diffuse social contacts are roughly equally important to disease spread, and that an effective lockdown must target both. We examine the cost–benefit of replacing a lockdown with tracing and quarantining contacts of the infected. Quarantine can contribute substantially to mitigation, even if it has short duration and is done within households. When reopening society, testing and quarantining is a strategy that is much cheaper in terms of lost workdays than a long lockdown. A targeted quarantine strategy is quite efficient with only 5 days of quarantine, and its effect increases when testing is more widespread.

## Introduction

The 2020 coronavirus (COVID-19) pandemic has raised the need for mitigation efforts that could reduce the peak of the epidemic^1,2^. To fulfill this need, theoretical modelling can play a crucial role. Traditional epidemiological models that assume universal or constant infection parameters are not sufficient to address case specific strategies like contact tracing. Therefore, we have developed an agent-based epidemiological model which takes into account that disease transmission happens in distinct arenas of social life that each play a different role under lockdown: The family, the workplace, our social circles, and the public sphere. This subdivision becomes especially important when discussing such efforts as contact tracing. Using an estimated weight of social contacts within each of these four spheres^3^ we discuss the effect of various mitigation strategies.

At the time of writing, both classical mean field models^4,5^ and agent-based models^2,6,7^ of the COVID-19 epidemic have already been made. The models often assume contact rates and disease transmission to be stratified by age^3,8,9^. In our model, we focus on social and work networks. This will directly allow us to test the effectiveness of localized quarantine measures. In addition we allow a fraction of the contacts to be non-specific, representing random meetings.

Within families, several age groups may live together. At the same time, disease transmission within the family is probably the variable that is the most difficult to change through social distancing. Furthermore, there is doubt as to what extent children carry and transmit the disease^10^. By ignoring age as a factor our agent-based model implicitly weights children on equal footing with anyone else, and our model is not designed to address scenarios where one specifically targets older people.

Analysing what role each area of social life plays also allows us to separately treat leisure activities, and since these play a smaller economic role than work, they may be restricted with a smaller toll on society. Furthermore, if widespread testing and contact tracing is implemented, a compartmentalisation like the one we are assuming here will help in assessing which people should be quarantined and how many will be affected at any one time.

In the following, we will investigate two closely related questions. First, how a lockdown is most effectively implemented, and second, how society is subsequently reopened safely, and yet as fast as possible. To answer the first, we must examine the relative effects of reducing the amount of contacts in the workplace, in public spaces, and in closely connected groups of friends. For the second question, we will look for viable strategies for mitigation that do not require a total lockdown. Here, we will focus on the testing efficiency and contact tracing^11^. Our results will hopefully be helpful in informing future containment and mitigation efforts.

**Figure 1:**
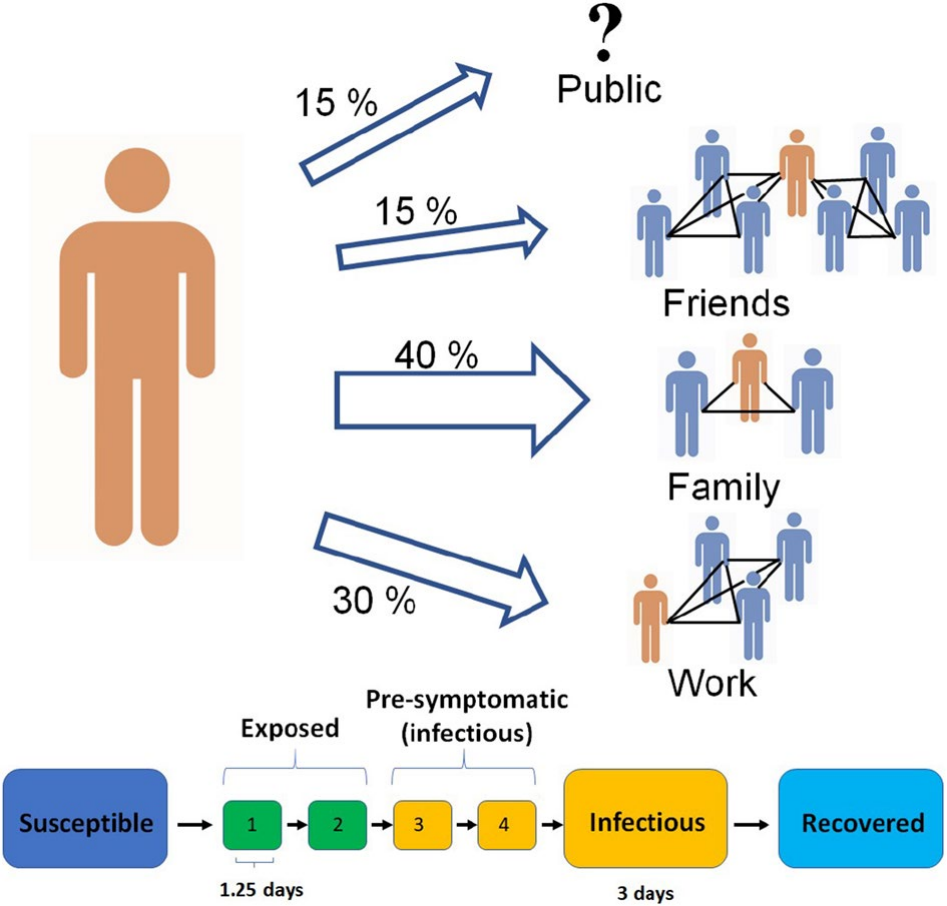
A diagram of the model structure. Each agent has a network consisting of a family, a workplace and two groups of friends. The family accounts for 40% of interactions. Work accounts for 30% and socialisation with friends accounts for a further 15%. The members of each of these 3 groups are fixed throughout the simulation. Finally, 15% of interactions happen “in public”, which we implement as an interaction with a randomly chosen other agent. Everyone in the work and friend sub-graphs are assumed to be connected to each other. Below the graph, the underlying mechanisms of the disease are shown. We divide the exposed state into four in order to get a more naturalistic gamma distribution of incubation periods. The two last exposed states are infectious, but asymptomatic, meaning that individuals will not get tested. This is to include presymptomatic infection. In our simulation we set the family groups to an average of 2 people, and the work network to 10 completely interconnected people. The friend network consists of two groups with five in each.

## Methods

Our proposed model divides social life into family life which accounts for 40% of all social interactions, work life accounting for 30%, social life in fixed friend groups accounting for 15%, and public life which accounts for another 15%^3^. Fig. 1 shows a schematic representation of the model. Interactions in public are taken to be completely random and not dependent on factors such as geography, density or graph theoretical quantities. Within families, workplaces, and friend groups, everyone is assumed to know everyone. Each agent is assigned one family and workplace, as well as two groups of friends. Workplaces on average contain ten people, whereas each friend group on average contains five. In the simulation runs presented here, we use a population of *N* = 5000 agents. Increasing the number of agents changes the outcome very little, except for minimising stochastic noise. We also do not allow migration in or out of the system.

We use a discrete-time stochastic algorithm. At each time-step (0*.*5 days), each person has one interaction with some other person. A “die roll” decides whether the person will interact with family, friends, work, or the public. The respective odds are the above-mentioned percentages 40:30:15:15. If the public is chosen, an entirely random person is selected, otherwise a person is drawn from a predefined group (family etc.). For each interaction, an infectious person has a fixed probability of passing on the disease to the person they interact with.

The family size distribution of is based on the distribution of Danish households^12^. The average number of people per household is approximately 2, and large households of more than 4 people have been ignored, as they account for less than 10% of the population. We believe that in a country where family sizes are larger and there are fewer singles, the family would be more important to the spread of disease. We test the effect of larger families in the supplement (Figs. S1–S3) and find that it does not change our overall conclusions.

We simulate the progression of disease using an SEIR model with four exposed states, *E* = *E*_1_ + *E*_2_ + *E*_3_ + *E*_4_, each lasting on average 1.25 days, corresponding to a mean incubation period of 5 days. The exposed states are presymptomatic, meaning that people will not get tested in the incubation period. We let stages E3;4 be as infectious as the I-stage, as data suggest that a substantial fraction of COVID-19 transmission happens before the onset of symptoms^13^. Multiple exposed states are included in order to get a naturalistic distribution of incubation periods. Li et al.^10^ report that the mean incubation period is approximately five days and the reported distribution is fitted well by the gamma distribution we obtain from our four E-stages.

A further problem is the duration of the infectious period (I). Viral shedding has been observed to last up to eight days in moderate illness^14^. On the other hand, according to Linton et al*.*^15^, the median time from onset to hospitalisation is three days. A bedridden patient (even if not hospitalised) is likely to transmit the disease less. To fit the observed mean serial intervals of 4.6 days of Nishiura et al*.*^13^ we model the infectious period as a single state with an average duration of three days. In addition, the infectious presymptomatic period lasts on average 2.5 days. In comparison Ref.^16^ uses a serial interval distribution with mean of 6.5 days. Other authors have suggested a longer serial interval^10^ with presymptomatic infections.

Finally, the transmission rate of the disease is estimated from an observed rate of increase of 23% per day in fatalities in the USA. This also fits the observation of a growth rate of ICU admissions of about 22.5% per day in Italy^17^. With our parameters this is reproduced by a basic reproduction number *R*_0_ ~ 3 (as we allow transmission in both directions when selecting two people, this is simulated by a rate for transmission of 0.2 per encounter). Li et al*.*^10^ estimate *R*_0_ at 2.2 based on a growth rate of 10% per day in confirmed COVID-19 cases in Wuhan prior to Jan. 4.

Having calibrated the model in this way, we want to explore mitigation strategies for the corona epidemic. Specifically, we will investigate the relative importance of the areas of social life, and the extent that reducing workplace size reduces disease spread. Moreover, we will examine the possible gain and cost by simple contact tracing and light quarantine practices.

## Results: mitigation strategies

**Figure 2:**
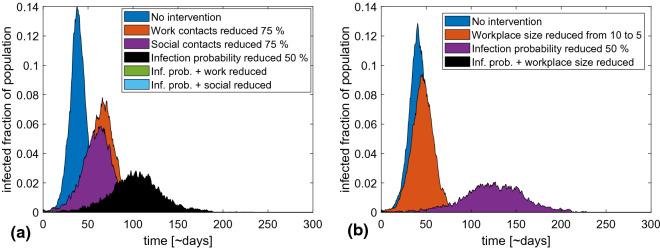
Comparison of various strategies with and without a reduction in transmission probability per encounter. (**a**) Reducing contacts in different social contexts by 75% through a lockdown. It can be seen that work and social contacts play roughly the same part in disease transmission. A reduction of infection probability makes the strategies relatively more effective. Reducing work contacts, for example, reduces the peak height by roughly 40% (relative to no intervention) if infection probability is high. If the infection probability is lowered, the strategy completely eliminates the epidemic. The apparent missing graphs in the figure are due to the epidemic dying out completely when a lockdown is combined with hygienic measures. (**b**) A similar comparison of the effects of reducing workplace sizes by half. This strategy is also relatively more effective if infection probability is reduced. The strategy also eliminates the epidemic at a lowered infection probability versus only a 30% reduction if infection probability remains high.

To illustrate the relative importance of the workplace and public life, we consider the scenarios in Fig. 2a. In the first scenario, nothing is done. In the second, contacts within the workplace are reduced by 75%, while in the third, contacts with friends and the public are reduced. Finally, we compare these with similar scenarios, but where good hygiene or keeping a distance reduces the probability of infection from all types of encounters by half.

In the figure, we see that the effects of reducing workplace and social contacts are roughly of the same magnitude. This reflects the assignment of 30% weight to each of these contact types. The slightly larger effect of social contacts reflects our assumption that these connections are less clustered than the workplace network. The two latter graphs show the scenarios where we both reduce infection probability within one group by 75% and overall infection probability by 50%. They show that an effective lockdown requires both restrictions of the time spent in the workplace and in the public sphere, and measures that reduce infection probability by increased hygiene and physical distancing.

The above results provide one useful piece of information. If the effect of workplace and social contacts are of the same order, it is of little importance which one is restricted. Ideally, both will be restricted for a period. However, when restrictions need to be lifted, authorities will primarily be able to control the workplace, whereas the social sphere relies on local social behavior. Obviously, it is economically more sustainable to lift the one with the largest social consequences first, by allowing people to return to work while encouraging keeping social gatherings at a minimum.

If restrictions are lifted before a substantial level of immunity is achieved, the epidemic will re-ignite. Therefore, we now examine what can be done to minimise spread in the reopened workplaces.

One possible strategy is to reduce the number of people allowed at any one time in each workplace. In Fig. 2b, we compare an epidemic scenario where the average number of employees per workplace is 10 with an epidemic where this number is reduced to 5. We further assume that the number of contacts per coworker remains the same, meaning that the number of contacts per person drops when workplace size is reduced.

It can be seen that fragmentation of physical spaces at workplaces could have a significant effect on the peak number of infected. In a situation with a risk of straining the healthcare system, this could be part of a mitigation strategy. Once again, the strategy becomes relatively more effective if the infection probability per encounter is also reduced. Compared to the cases with no workplace size reduction, making workplaces smaller leads to a greater relative reduction in peak size if infection probability is lower, completely eliminating the epidemic at an infection probability reduction of 50%.

A more local strategy that can be employed when reopening society is widespread testing and contact tracing. As mentioned above, Hellewell et al*.*^11^ have suggested that this can be effective in containing COVID-19 outbreaks provided high efficiency in detecting infected individuals. Contact tracing has previously been modeled in relation to other epidemics^18^, and used successfully against smallpox^19^ and SARS^20^.

One obstacle to the widespread implementation of this strategy is the difficulty of tracing contacts. Therefore, we will here implement a crude form of contact tracing where we (1) close the workplaces of people who are tested positive for the disease, (2) isolate their regular social contacts for a limited period, and (3) keep symptomatic individuals in quarantine until they recover. We will see that such a 1 step tracing and quarantine strategy (1STQ) can give a sizeable reduction in disease spread while costing fewer lost workdays than overall lockdown. Our simulations include the limitations imposed by not being able to trace the estimated 15% of infections from random public transmissions. Thus, the strategy does not require sophisticated contact tracing but could be implemented based on infected people being able to recollect their recent face-to-face encounters with friends.

It should be noted that we here quarantine persons in their own households, thereby making our contact tracing strategy easier to implement in practice. In particular, family members of a quarantined person are still free to interact outside their home if they are not themselves tested positive. The drawback of such light quarantine practices is that infected persons in quarantine may still transmit the infection to their families.

**Figure 3:**
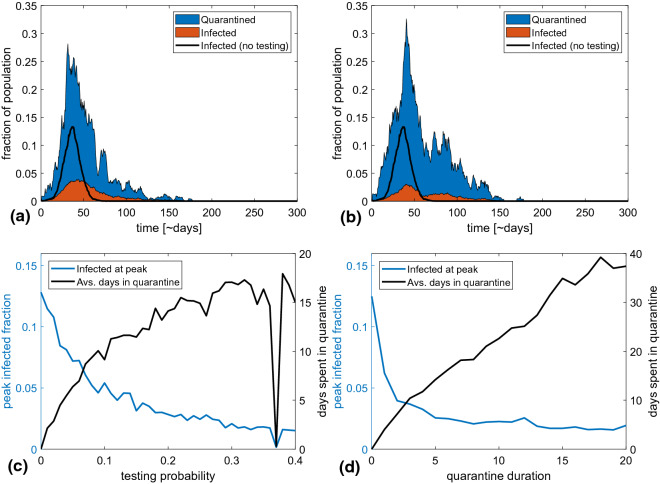
The effect of quarantine duration and testing probability. (**a**,**b**) show examples of epidemic trajectories for a quarantine length of 5 days and a daily testing probability of 10 and 20% respectively. The blue section of the curve shows the fraction of people who are in quarantine but healthy or presymptomatic, while the orange section shows the fraction who are ill. (**c**,**d**) show the peak fraction of population infected (left y-axis) and time spent in quarantine (right y-axis) as a function of testing chance and quarantine length. The number of days in quarantine was calculated using our standard group sizes which connect each person to approximately 20 others. The average quarantine time scales proportionally with this assumed connectivity. (**c**) With a quarantine length of 5 days, it is possible to reduce the peak number of infected by eight percentage points, corresponding to a 60% drop, if the probability of infected people being tested is only 10% per day of illness. However, the price of this is that each person is on average quarantined once during the epidemic. If testing is more widespread, the epidemic peak can be further reduced, until it finally becomes unstable at a testing probability of around 40% per day. (**d**) Epidemic peak and time spent in quarantine as a function of quarantine length for a testing probability of 20% per day. The average time spent in quarantine increases linearly with the length of quarantine. On the contrary, the effect of quarantine on the peak height appears to stagnate at approximately 5 days.

Figure 3 examines how increased testing efficiency systematically improves our ability to reduce the peak disease burden. This would then be a more cost efficient way to mitigate the pandemic than a complete lockdown where each person would lose several man-months. Even detecting as little as 5% of COVID-19 infected per day (which with an average symptomatic disease duration of 3 days corresponds to finding approximately 15% of the infected) can potentially reduce the peak number of cases by 50%. If 10% efficiency is possible, corresponding to detecting about a third of infectious cases, then peak height could be reduced by a factor of almost three with less than two weeks in quarantine per person during the entire epidemic. This is illustrated in Fig. 3a where peak height is reduced from 0.13 to 0.04 at 10% testing efficiency.

The main cost of the quarantine option is the quarantine time. Figure 3d examines the efficiency versus cost of as a function of quarantine length. It can be seen that there is little gain in extending the quarantine period beyond the 5-day duration of the incubation period. For this reason we opted for 5 days in quarantine in panel (a, b). As a consequence, an average person will stay around 12 days in quarantine during the course of the epidemic with a testing probability of 10% per day. This time can be reduced if people can be convinced of smaller work environments and fewer face-to-face contacts per week. Fragmentation of our networks into smaller groups will reduce both quarantine overhead and the direct transmission of the disease (Fig. 2b, orange curve).

A prolonged lockdown will hugely disrupt society, and it is questionable whether a complete eradication of the virus is possible anyway. Therefore, most governments have aimed at softening the epidemic curve, with varying degrees of success. The one step contact tracing with testing and quarantine is a means to this end and would work most effectively in combination with other efforts to reduce *R*_0_.

Finally, we investigate whether an aggressive testing and contact tracing strategy could work if implemented at a late stage in an epidemic. This could be relevant if for example the strategy is part of an effort to reopen society after a period of lockdown.

**Figure 4:**
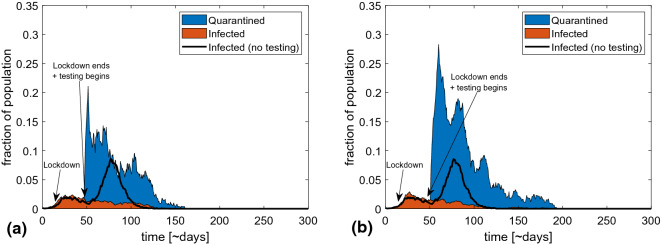
Various trajectories of the epidemic when combining a lockdown and a late-onset 1STQ strategy. (**a**) shows a possible course of an epidemic where restrictions in public and work life (by 75%) are implemented when 1% are infected and lifted after 30 days, being replaced by a testing and tracing regime with a testing probability of 20% per day and quarantine duration of 5 days. The black line shows the fraction of infected if no testing is implemented. We see that this level of testing and quarantine is sufficient to prevent a resurgence of the epidemic. (**b**) is similar, but here quarantine lasts 10 days. This is about as effective as (**a**) but costs a lot more in terms of number of people in quarantine.

In Fig. 4, we show two possible scenarios where testing and contact tracing is implemented after a 30-day lockdown with a 75% reduction of the work and social spheres. The lockdown is initiated when 1% of the population is infected. In (a) we subsequently test and quarantine the infected and their contacts for 5 days, while in (b) the required quarantine is set to 10 days. We assume a testing efficiency of 20% chance of detection for each day a person is symptomatic. The progression of the epidemic without testing is marked by a black graph for comparison.

From the figure one sees that the strategy of even relatively short quarantines also works with a late onset. At a realistic detection probability, it prevents a resurgence of the epidemic. Nonetheless, it is quite costly initially, with a very high peak in number of quarantined people. Importantly, the effect does not increase with a longer quarantine period, but the cost is substantially larger.

## Discussion

Pandemics such as the one caused by COVID-19 can pose an existential threat to our social and economic life. The disease itself is serious and leaves specific epidemic signatures and characteristics that make traditional contact tracing difficult. In particular it is highly infectious, can sometimes be transmitted already two days after exposure, and a large fraction of transmission happens before the onset of symptoms. As such it is difficult to contain without a system-wide lockdown of society. Nonetheless, a successful containment in South Korea used contact tracing. This motivated us to explore a one-step contact tracing/quarantine strategy (1STQ).

Using reasonable COVID-19 infection parameters we find that the 1STQ strategy can contribute to epidemic mitigation, in the sense that it can reduce the peak number of infected individuals by about a factor of two even with a realistic testing rate of 10% per day of illness. This was illustrated systematically in Fig. 3. The main cost was people in self-quarantine and not contributing to the workforce. In comparison one has to consider that a society-wide lockdown with similar reduction in peak height would have to last for about 100 days (see Fig. 2). Thus, the lockdown would require of order 100 days of quarantine (or at least extensive social distancing) per person, whereas testing and isolation only requires on average around 15 days per person with a 5-day quarantine even at high testing probabilities. Importantly these numbers can be reduced if people are able to lower their number of contacts.

A noticeable objection to the 1STQ strategy is the fraction of cases with so weak symptoms that people do not contact health authorities. The effect of such limitations is in our model parameterized through the detection probability. From Fig. 3c one sees that when the detection probability goes below 3% (a rate of 1% per day) the peak reduction of the 1STQ strategy becomes only of the order 1 percentage point. It should also be noted that, since we rely on symptoms to determine who stays in quarantine, and people in the infectious/symptomatic stage are assumed to always stay in quarantine, we implicitly assume that all infected persons develop at least some symptoms at some point. This may be a break from reality.

The increasing availability of tests may also change the perspectives of the 1STQ strategy. With widely available rapid tests, it will be possible to test everyone regularly, and to test all quarantined persons before they leave quarantine. Supplementary Figure S4 deals with the results of such a testing strategy and finds that it makes it possible to totally control the epidemic, or to mitigate it without quarantining any healthy individuals. To put this into perspective, the drawbacks of widespread, but slow testing is examined in the supplementary Fig. S5. Here, we find that the 1STQ strategy is most efficient with no test delay, and that delayed contact tracing is comparable to a primitive lockdown.

One interesting point which we have not examined here, is that real-world social networks are heterogeneous, with a large variance in number of contacts. It may be expected, for example, that workers in customer-facing positions in shops will have a high risk of catching the disease and passing it on. The effects of this heterogeneity is examined more closely in Ref.^21^ Here, it is concluded that heterogeneity in the number of contacts enhances the effect of contact tracing, since persons with many contacts are both more likely to pass on the disease and more likely to be quarantined.

In Ref.^11^, the authors suggest a 1STQ strategy similar to the one we here model. The main points of the present analysis is the focus on mitigating instead of eradicating the epidemic, our suggestion of a shorter quarantine length, and the implementation of quarantine together with other members of the household instead of total isolation. Our stochastic, agent-based approach also allows for local failures due to the limited duration of quarantine (people may not yet be symptomatic when exiting quarantine) and the non-traceable public contacts (set to 15%).

Finally, one noticeable finding is that contact tracing and reduction of contacts per person is still feasible even at a later stage of the epidemic. As can be seen in Fig. 4, a lockdown and subsequent reopening with testing and contact tracing is highly effective in controlling the epidemic. Our study that lockdowns have an important role to play in epidemic mitigation, but that they can be replaced by a 1STQ strategy once the epidemic is under control.

The COVID-19 pandemic has set both governments, health professionals, and epidemiologists in a situation that is more stressful and more rapidly evolving than anything in recent years. Due to the uncertainties caused by a situation in flux, it is difficult to predict anything definite about what works and what does not. The empirical observation that lockdowns worked in both China, and in a milder form in Denmark shows that our assumption of a 75% reduction in specific infection rates under lockdown is realistic. Our main result is that some of these restrictions can be replaced by testing, one-step contact tracing and short periods of quarantine. This is far cheaper than total lockdowns. Perhaps most importantly, these measures work best in combination. As is highly relevant to the current epidemic stage of COVID-19, we pinpoint that 1STQ can be successfully implemented also at a late stage of the epidemic where testing may become massively available.

## Supplementary information

Supplementary Information.

## Data Availability

Plots of alternative variants of our model (including alternative testing strategies and larger family sizes) can be found in the supplementary material. The code used to produce the plots shown in this article is available on Figshare under the URL https://doi.org/10.6084/m9.figshare.12206735.v4.
