# Supplementary material for: Cost–benefit of limited isolation and testing in COVID-19 mitigation
Source: Sci Rep. 2020 Oct 29;10:18543. doi: 10.1038/s41598-020-75640-2 (PMC7596701; doi:10.1038/s41598-020-75640-2)
Supplement: Supplementary file 1 — Supplementary Information. [file 41598_2020_75640_MOESM1_ESM.pdf]

Supplementary figures for the article "Cost-benefit of limited isolation and testing in COVID-19 mitigation"

Andreas Eilersen[1,*,+] **and Kim Sneppen**[1,+]

[1]University of Copenhagen, Niels Bohr Institute, 2100 København Ø, Denmark
[*]andreaseilersen@nbi.ku.dk
[+]these authors contributed equally to this work

The effects of family size

To examine the effect of the family size distribution on our conclusions, we have repeated the figures of the main article with twice as large families. That is to say, single households now contain two people, previously two-person households contain four etc. We see that while this somewhat affects the shape of the epidemic curve, it does not significantly influence any of our conclusions.

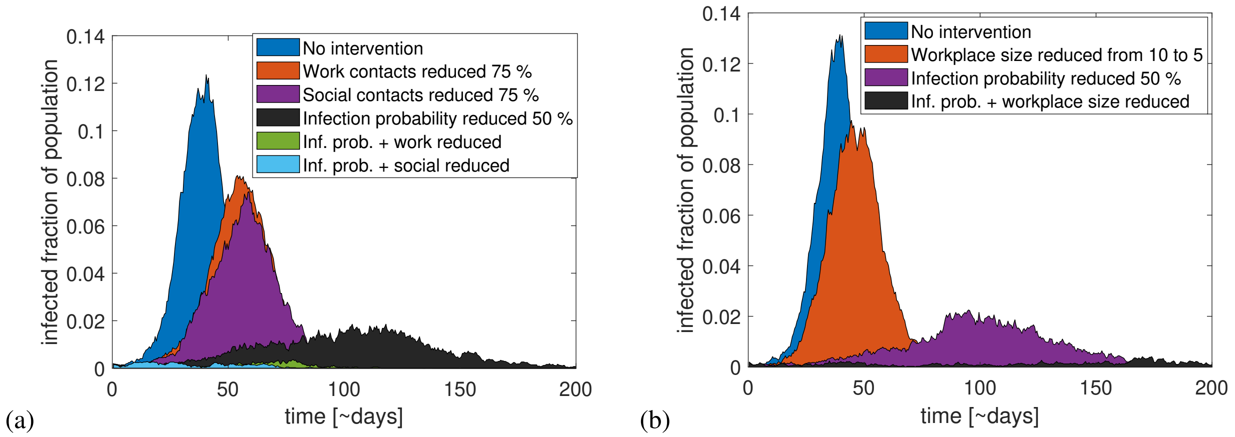

Figure S1. (a) A comparison of various containment strategies that include a lockdown reducing social or work contacts by 75 %. We see that if we combine these measures with improved hygiene or social distancing, reducing the transmission risk per encounter by half, this is sufficient to stop the epidemic completely. This is the same conclusion as in the main article. (b) The effect of reducing workplace sizes by half, from 10 to 5 people per workplace on average. As in the main article, this has a significant effect on the epidemic, and in this version of the model, it is also enough to fully mitigate it if combined with hygienic measures that halve the infection probability.

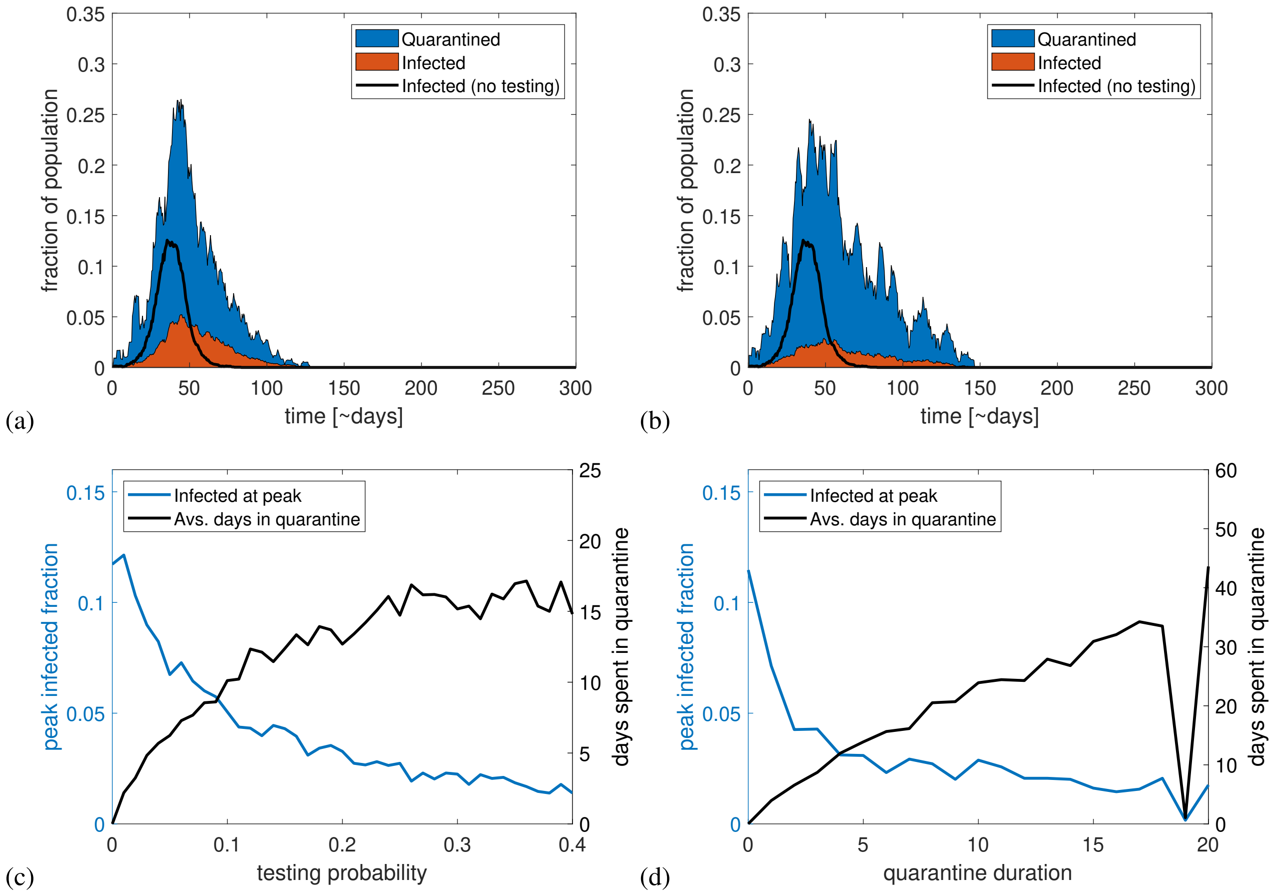

Figure S2. (a) and (b) show epidemic trajectories and fraction of people in quarantine for testing probabilities of 10 and 20 % per day respectively. The black line shows the epidemic trajectory in the absence of testing and contact tracing. (c) shows the infected fraction of the population at the peak of the epidemic (left axis) and the average number of days each person spends in quarantine during the epidemic as a function of daily testing probability while symptomatic. (d) shows the same variable but as a function of quarantine duration. It can be seen here as well that little is gained from quarantine beyond five days. When quarantine lasts longer than about 18 days, the epidemic becomes unstable, sometimes dying out.

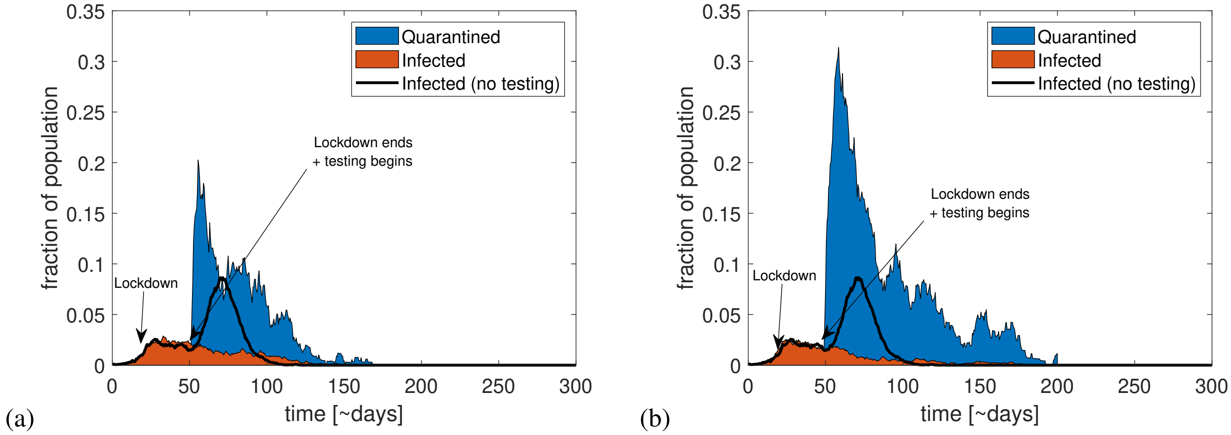

Figure S3. Two examples of epidemic trajectories when combining a lockdown with a 1STQ strategy. When 1 % of the population is infected, a lockdown is implemented, reducing social and work contacts by 75 %. It is lifted after 30 days and replaced by a testing and contact tracing strategy with a daily testing probability of 20 % for symptomatic individuals. The duration of the quarantine in panel (a) is five days and in (b) it is ten days. We see that the longer quarantine does not change the effect on the epidemic much, but it does increase the number of people in quarantine.

Weekly tests

In this section, we let all agents get tested regularly at a one-week interval in addition to a 20 % daily testing probability for symptomatic individuals. This strategy will become increasingly feasible with increasing availability of rapid tests. It can be seen that weekly tests are sufficient to contain the epidemic if contacts of the infected are quarantined (fig. S4(a)). If only the infected themselves are quarantined, it is still enough to mitigate the epidemic as seen in panel (b), giving us a situation where no healthy persons are unnecessarily quarantined. With as widespread testing as this, it is assumed that everyone is tested before leaving quarantine, meaning that no presymptomatic individuals are let out of quarantine.

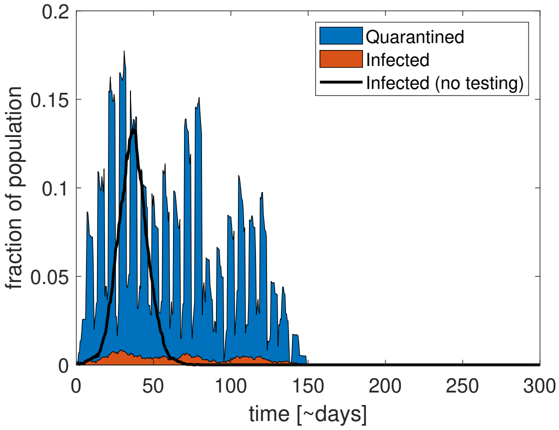

(a)

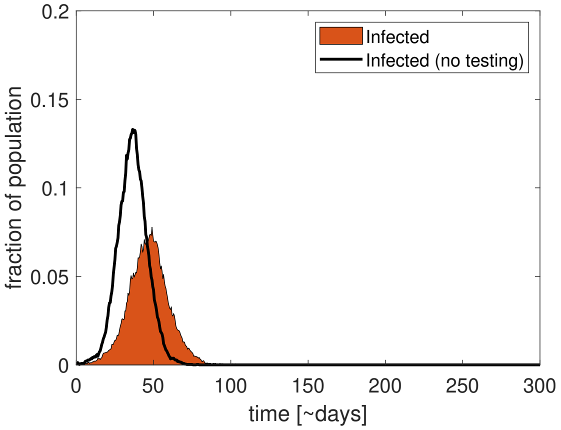

(b)

Figure S4. (a) Infected and quarantined fraction of the population if everyone, in addition to tests of the symptomatic, take one weekly test, thus also discovering any presymptomatic cases. This is enough to keep the epidemic in check. In (b) the same strategy is followed, but only the infected themselves are quarantined. This still significantly reduces the epidemic peak. The unmitigated epidemic trajectory is shown by the black curves.

Delayed test results

In the following, we examine the effect of a delay in obtaining test results. We assume that people who are tested have to wait for a number of days before getting the result, thus delaying contact tracing and quarantine. We find that the effect of contact tracing only subsides slowly with increasing test delay, as seen in fig. S5. This we believe is due to the effect of the large number of people in quarantine at the peak of the epidemic. Even though the contact tracing efforts are less effective when test results are delayed, having a large fraction of the population in quarantine works as a primitive lockdown, lowering the epidemic peak by "brute force".

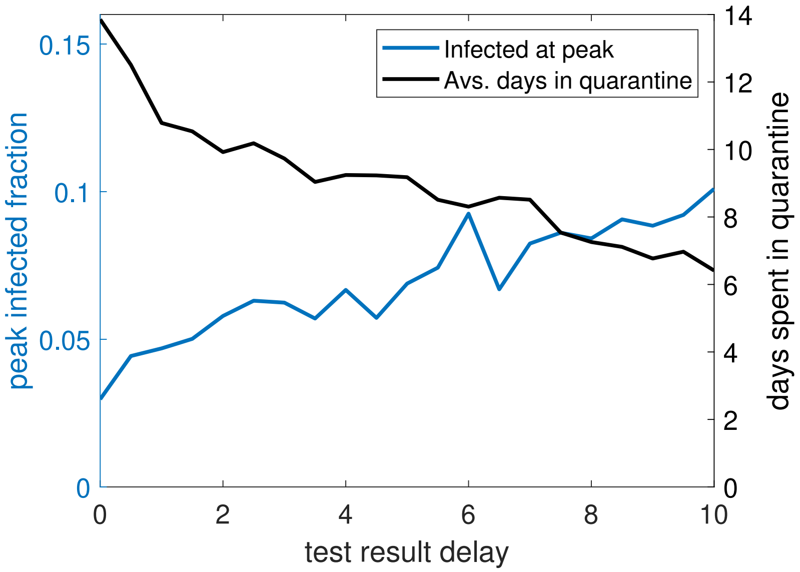

Figure S5. This figure shows the effect of delayed test results on the peak fraction of infected and the average number of days spent in quarantine during the epidemic. We see that the peak fraction of infected grows linearly with the delay, until the effect is nearly gone after ten days. At the same time, the amount of time spent in quarantine decreases. Part of the mitigation at long delays is expected to stem from the fact that a significant portion of the population is quarantined, and therefore less infectious, at all times. The testing probability is here set to 20 % per day of symptomatic illness and the quarantine length is set to five days. The testing delay is assumed to not affect people's ability to leave quarantine.
